# Myxobacteria-Derived Outer Membrane Vesicles: Potential Applicability Against Intracellular Infections

**DOI:** 10.3390/cells9010194

**Published:** 2020-01-12

**Authors:** Adriely Goes, Philipp Lapuhs, Thomas Kuhn, Eilien Schulz, Robert Richter, Fabian Panter, Charlotte Dahlem, Marcus Koch, Ronald Garcia, Alexandra K. Kiemer, Rolf Müller, Gregor Fuhrmann

**Affiliations:** 1 Helmholtz Centre for Infection Research (HZI), Biogenic Nanotherapeutics Group (BION), Helmholtz Institute for Pharmaceutical Research Saarland (HIPS), Campus E8.1, 66123 Saarbrücken, Germany; adriely.goes@helmholtz-hips.de (A.G.); philipp.lapuhs@gmail.com (P.L.); thomas.kuhn@helmholtz-hips.de (T.K.); eilien.schulz@helmholtz-hips.de (E.S.); 2 Department of Pharmacy, Saarland University, Campus Building E8.1, 66123 Saarbrücken, Germany; Robert.Richter@helmholtz-hips.de (R.R.); rolf.mueller@helmholtz-hips.de (R.M.); 3 Helmholtz Centre for Infection Research (HZI), Department of Drug Delivery (DDEL), Helmholtz Institute for Pharmaceutical Research Saarland (HIPS), Campus E8.1, 66123 Saarbrücken, Germany; 4 Helmholtz Centre for Infection Research (HZI), Department of Microbial Natural Products (MINS), Helmholtz Institute for Pharmaceutical Research Saarland (HIPS), Campus E8.1, 66123 Saarbrücken, Germany; fabian.panter@helmholtz-hips.de (F.P.); ronald.garcia@helmholtz-hips.de (R.G.); 5 Department of Pharmacy, Pharmaceutical Biology, Saarland University, 66123 Saarbrücken, Germany; charlotte.dahlem@uni-saarland.de (C.D.); pharm.bio.kiemer@mx.uni-saarland.de (A.K.K.); 6 INM-Leibniz Institute for New Materials, Campus D2 2, 66123 Saarbrücken, Germany; marcus.koch@leibniz-inm.de; 7 German Center for Infection Research (DZIF), 38124 Braunschweig, Germany

**Keywords:** extracellular vesicles, antimicrobial resistance, *Staphylococcus aureus*, intracellular infection, outer membrane vesicles, biogenic drug carriers

## Abstract

In 2019, it was estimated that 2.5 million people die from lower tract respiratory infections annually. One of the main causes of these infections is *Staphylococcus aureus*, a bacterium that can invade and survive within mammalian cells. *S. aureus* intracellular infections are difficult to treat because several classes of antibiotics are unable to permeate through the cell wall and reach the pathogen. This condition increases the need for new therapeutic avenues, able to deliver antibiotics efficiently. In this work, we obtained outer membrane vesicles (OMVs) derived from the myxobacteria *Cystobacter velatus* strain Cbv34 and *Cystobacter ferrugineus* strain Cbfe23, that are naturally antimicrobial, to target intracellular infections, and investigated how they can affect the viability of epithelial and macrophage cell lines. We evaluated by cytometric bead array whether they induce the expression of proinflammatory cytokines in blood immune cells. Using confocal laser scanning microscopy and flow cytometry, we also investigated their interaction and uptake into mammalian cells. Finally, we studied the effect of OMVs on planktonic and intracellular *S. aureus*. We found that while Cbv34 OMVs were not cytotoxic to cells at any concentration tested, Cbfe23 OMVs affected the viability of macrophages, leading to a 50% decrease at a concentration of 125,000 OMVs/cell. We observed only little to moderate stimulation of release of TNF-alpha, IL-8, IL-6 and IL-1beta by both OMVs. Cbfe23 OMVs have better interaction with the cells than Cbv34 OMVs, being taken up faster by them, but both seem to remain mostly on the cell surface after 24 h of incubation. This, however, did not impair their bacteriostatic activity against intracellular *S. aureus*. In this study, we provide an important basis for implementing OMVs in the treatment of intracellular infections.

## 1. Introduction

Pulmonary infections represent a serious health risk for today’s society. The Global Burden of Disease Study 2016 has estimated that lower respiratory infections are one of the leading causes of death worldwide, especially in children aged five years and younger [1,2]. Lung infection is also a frequent complication for patients with cystic fibrosis (CF), an inherited, systemic disorder caused by a mutation in the cystic fibrosis trans-membrane regulator channel [3,4]. CF lung disease is characterized by the accumulation of a thick mucus in the airway, which favors lung inflammation and persistent, chronic bacterial infection. One of the main pathogens causing lung infections in CF patients is *Staphylococcus aureus* (*S. aureus*) [5,6]. Besides being able to form biofilms, it is known that *S. aureus* can also invade professional and non-professional phagocytes and is able to survive intracellularly by escaping the endosomal pathway into the cytoplasm [7,8,9,10].

The antibiotics currently available on the market are not optimal for treating intracellular infections, as most of them need higher concentrations and a longer therapy time to induce a positive effect [11]. Generally, free antibiotics (e.g., aminoglycosides) are unable to eradicate intracellular infections due to their hydrophilic characteristics and high polarity, which prevent their permeation into mammalian cells [12,13,14,15,16,17]. To address this problem, increased efforts have been made towards improved drug delivery using nanotechnology, surface modification, biomimetic and biogenic carriers to overcome this barrier [18,19,20,21]. Carriers such as liposomes have been successful at delivering antibiotics to biofilms and eradicating them [22].

Myxobacteria are a group of Gram-negative bacteria that are abundant in soil. Many of these bacteria show predatory behavior [23], and interact, move and prey by forming coordinated swarms [24]. They belong to the class Delta Proteobacteria, phylum Proteobacteria. Myxobacteria are potent producers of antimicrobial compounds [25,26,27,28] and they are non-pathogenic to humans. Outer membrane vesicles (OMVs) are nanoparticles shed from the outer membrane of Gram-negative bacteria [29,30,31]. OMVs derived from myxobacteria have been shown to be involved in intercolony communication but also as predatory weapons against other bacteria [32]. We recently reported on myxobacterial OMVs with inherent antimicrobial properties due to their cystobactamid cargo [33]. Cystobactmids are topoisomerase inhibitors that have potent antibacterial activity [34]. However, the antimicrobial activity of myxobacterial OMVs has only been shown against the planktonic model bacterium (*Escherichia coli* strain DH5-alpha), which is not clinically relevant. Here, we expand the evaluation of these OMVs to clinically important *S. aureus* pathogens. For potential OMV translation, it is necessary to biotechnologically obtain them at large amounts. Myxobacterial cultures are suitable for this purpose, because they can be increased to several liters, which facilitates the large-scale isolation of their OMVs [34].

In this study, we explore the myxobacterial strains *Cystobacter velatus* Cbv34 and *Cystobacter ferrugineus* Cbfe23 for the production of natural antibacterial OMVs and analyze their potential for uptake by mammalian cells and the eradication of intracellular *S. aureus*. Our results show that both Cbv34 and Cbfe23 OMVs are efficiently taken up into macrophages and epithelial cells without affecting their viability. Importantly, they presented an antibacterial effect against intracellular *S. aureus*.

## 2. Materials and Methods

### 2.1. Myxobacterial Culture

The myxobacterial strains Cbv34 (*Cystobacter velatus*) and Cbfe23 (*Cystobacter ferrugineus*) were cultured in 100 mL M-Medium (*w*/*v*, 1.0% phytone, 1.0% maltose, 0.1% CaCl_2_, 0.1% MgSO_4_, 50 mM HEPES, pH adjusted to 7.2 with KOH) at 30 °C and 180 rpm. Upon reaching the stationary phase at 6–7 days, 50 mL of the culture was removed for OMV isolation. The remaining volume was used as an inoculum for the next passage of the myxobacterial culture. Growth curves of the cultures were established as described in a previous study [33].

### 2.2. Isolation and Purification of Outer Membrane Vesicles

In order to obtain potent OMVs with a high yield, the myxobacteria were cultured for at least three passages prior to performing the isolation. Fifty milliliters of the cultures were used and centrifuged at 9500 × *g* for 10 min at 4 °C. The supernatant was transferred to a new falcon tube and centrifuged once again at 9500 × *g*, for 15 min at 4 °C. Thirty milliliters of the resulting supernatant was added into an ultracentrifugation tube and pelleted at 100,000 × *g* for 2 h at 4 °C using a rotor type SW 32 Ti (Beckman Coulter). The supernatant was removed, and the pellet was dispersed in 300 μL phosphate buffered saline (PBS, Gibco PBS tablets without calcium, magnesium and phenol red) (Sigma-Aldrich; Co., St. Louis, MO, USA) filtered with 0.2 µm mixed cellulose ester filters (Whatman, GE Healthcare UK Limited, Little Chalfont, UK). In order to remove the free protein present in the pellet, a size exclusion chromatography (SEC) was performed. The pellet was added to a 60 mL column filled with 35–40 mL of Sepharose CL-2B (GE Healthcare Bio-Sciences AB, Uppsala, Sweden) in PBS. One milliliter fractions of OMVs in PBS were collected into polypropylene (PP) tubes (Axygen, Corning Incorporated, Reynosa, Mexico) next to a Bunsen burner, to obtain aseptic conditions. The fractions were kept at 4 °C for up to one month. Prior to infection, experiments and measurements of particle parameters, the fractions were filtered with Puredisc 25 AS (GE Healthcare UK Limited, Little Chalfont, UK) to assure sterility.

### 2.3. Liquid-Chromatography Coupled Mass Spectrometry

#### 2.3.1. OMV Preparation

OMV pellets were resuspended in 500 µL of particle-free PBS and lyophilized for 16 h. The dried pellet was mixed with 300 µL of MeOH and vortexed for 1–2 min. The OMV extract was centrifuged to remove debris. Then, the supernatant was transferred to a vial for LC-MS analysis.

#### 2.3.2. UHPLC MS Conditions

UPLC-hrMS analysis was performed on a Dionex (Germering, Germany) Ultimate 3000 RSLC system using a Waters (Eschborn, Germany) BEH C18 column (50 × 2.1 mm, 1.7 µm) equipped with a Waters VanGuard BEH C18 1.7 µm guard column. Separation of 1 µl sample was achieved by a linear gradient from (A) H_2_O + 0.1% FA to (B) ACN + 0.1% FA at a flow rate of 600 µL/min and a column temperature of 45 °C. Gradient conditions were as follows: 0–0.5 min, 5% B; 0.5–18.5 min, 5%–95% B; 18.5–20.5 min, 95% B; 20.5–21 min, 95%–5% B; 21–22.5 min, 5% B. UV spectra were recorded by a DAD in the range 200–600 nm. The LC flow was split to 75 µL/min before entering the Bruker Daltonics maXis 4G hrToF mass spectrometer (Bremen, Germany) using the Apollo II ESI source. Mass spectra were acquired in centroid mode ranging from 150–2500 *m*/*z* (mass/charge) at a 2 Hz full scan rate. Mass spectrometry source parameters are set to 500 V as end plate offset; 4000 V as capillary voltage; nebulizer gas pressure 1 bar; dry gas flow of 5 L/min and a dry temperature of 200 °C. Ion transfer and quadrupole settings are set to Funnel RF 350 Vpp; Multipole RF 400 Vpp as transfer settings and Ion energy of 5 eV as well as a low mass cut of 300 *m*/*z* as Quadrupole settings. Collision cell is set to 5.0 eV and pre pulse storage time is set to 5 µs. Spectra acquisition rate is set to 2 Hz. Calibration is done automatically before every LC-MS run by the injection of sodium formate and calibration on the sodium formate clusters forming in the ESI source. All MS analyses are acquired in the presence of the lock masses C_12_H_19_F_12_N_3_O_6_P_3_; C_18_H_19_O_6_N_3_P_3_F_2_ and C_24_H_19_F_36_N_3_O_6_P_3_, which generate the [M + H]^+^ ions of 622.03; 922.01 and 1221.10.

#### 2.3.3. LC-MS Data Bucketing and Annotation Using Metaboscape Software

Acquired LC-MS data are bucketed by Bruker Metaboscape 4.0 SR1 using the qTOF data optimized TReX 3D algorithm. Bucketed analyses are checked for library hits against our in-house database of myxobacterial natural products called myxobase. Library hits are retained if they show a retention time deviation under the described parameters of less than 0.2 min, *m*/*z* value deviation of less than 5 ppm and isotope pattern ratio congruence lower than 30 milliSigma.

#### 2.3.4. Conditions for MS² Analysis

LC and MS conditions for automatic precursor selection MS² data acquisitions remain as described in section-standardized UHPLC-MS conditions. CID Energy is ramped from 35 eV for 500 *m*/*z* to 45 eV for 1000 *m*/*z* and 60 eV for 2000 *m*/*z*. MS full scan acquisition rate was set to 2 Hz and MS/MS spectra acquisition rates were ramped from 1 to 4 Hz for precursor ion intensities of 10 to 1000 kcts.

#### 2.3.5. GNPS Clustering Parameters

MS^2^ data of the vesicle extracts were uploaded to the Global Natural Product Social Molecular Networking (GNPS) server at University of California San Diego [35]. A molecular network was created, using a parent mass tolerance of 0.05 Da and a fragment ion tolerance of 0.1 Da. Cosine score of edges considered to network was set to extend 0.7 and the minimum matched fragment peaks were set to 5. The clustered dataset was visualized using Cytoscape 3.7.2 (Cytoscape Consortium, UCSD San Diego, USA).

### 2.4. Cryogenic Electron Microscopy

Cryogenic transmission electron microscopy (cryo-TEM) was performed on OMVs pellets after ultracentrifugation and purified fractions as previously described [33]. Three to four microliters of the sample were dropped onto a holey carbon grid (type S147-4, Plano, Wetzlar, Germany) and plotted for 2 s before plunging into liquid ethane at T= −165 °C using a Gatan (Pleasanton, CA, USA) CP3 cryo plunger. The sample was transferred under liquid nitrogen to a Gatan model 914 cryo-TEM sample holder and analyzed at T = −173 °C by low-dose TEM bright-field imaging using a JEOL (Tokyo, Japan) JEM-2100 LaB6 at 200 kV accelerating voltage. Images with 1024 × 1024 pixels were acquired using a Gatan Orius SC1000 CCD camera at 2 s binning and 4 s imaging time.

### 2.5. Cell Culture

The murine macrophage cell line RAW 264.7 was obtained from the European Collection of Authenticated Cell Cultures (ECACC) and cultured in Dulbecco’s Modified Eagle Medium (DMEM) (1X) (Life Technologies Limited, Paisley, UK) supplemented with 10% (*v*/*v*) fetal bovine serum (FBS) (Life Technologies Limited, Paisley, UK). The adenocarcinomic human alveolar basal epithelial cell line (A549) and the human acute leukemia monocyte cell line (THP-1) were both purchased from the German Collection of Microorganisms and Cell Cultures (DSMZ) and cultured in RPMI 1640 (Life Technologies Limited, Paisley, UK) supplemented with 10% (*v*/*v*) FBS. RAW264.7 and A549 cells were split once a week, starting with 0.2 × 10^6^ cells/13 mL for RAW and A549. THP-1 cells were split twice a week to a seeding density of 2–3 × 10^6^ cells/13 mL. Mycoplasma tests were conducted regularly.

### 2.6. Cell Viability and Cytotoxicity

To assess the viability and cytotoxicity of cells upon treatment, the cells were seeded into 96-well plates at a density of 20,000 cells/well (RAW 264.7 and A549) and 100,000 cells/well (THP-1). To stimulate the THP-1 cells into macrophages, they were incubated with 30 ng/mL of phorbol 12-myristate 13-acetate (PMA). All cells were grown for 48 h. Within every set of experiments, a live-control, using cells treated with PBS (100 µL cell medium plus 100 µL PBS), which did not show any change in cell viability or morphology, and a dead-control, using cells treated with 1% (*v*/*v*) Triton-X 100 (Sigma-Aldrich; Co., St. Louis, MO, USA) were included. During the assays, cells were cultured in FCS-free medium to prevent falsified results caused by traces of lactate dehydrogenase (LDH) contained in the medium. Cells were incubated with 100 µL of OMV suspension in PBS at different concentrations (1 × 10^12^, 1 × 10^11^ and 1 × 10^10^ OMVs/mL) and 100 µL of cell medium for 24 h. For cytotoxicity evaluation, after an incubation time of 24 h, 100 µL of medium were removed to be analyzed by LDH-assay, which detects the amount of LDH released into the medium upon cell death after plasma membrane damage, and mixed with 100 µL of LDH-reagent (Roche Diagnostics GmbH, Mannheim, Germany), prepared according to the supplier’s protocol. After an incubation time of 5 min at room temperature (RT), the absorbance of the solution was measured at 492 nm. For viability, PrestoBlue (Thermo Fisher Scientific, Waltham, MA, USA) reagent, which detects metabolically active cells, was diluted by 1 in 10 with the respective medium of the cells. The remaining medium in the wells was removed and 100 µL of the diluted PrestoBlue reagent was added. After 20 min of incubation at 37 °C, fluorescence of the emerging dye was measured at an excitation of 560 nm and emission of 590 nm with a Tecan Infinity Pro 200 (Tecan, Männedorf, Switzerland). plate reader.

### 2.7. Cytokine Detection of OMV-Treated PBMCs

Peripheral blood mononuclear cells (PBMCs) were isolated by centrifugation from buffy coats, derived from three different donors obtained from the Blood Donation Center, Saarbrücken, Germany, authorized by the local ethics committee (State Medical Board of Registration, Saarland, Germany; permission no. 173/18). The cells were cultured in a 96-well plate with 100,000 cells per well in RPMI 1640 (Sigma Aldrich, St. Louis, MO, USA). Pellets of OMVs were isolated by ultracentrifugation as described above. One-hundred microliters of sterile PBS was used to resuspend the pellets. The particle concentration was determined via nanoparticle tracking analysis and the samples were diluted with PBMC medium to a final concentration of 5 × 10^6^ and 5 × 10^5^ particles per cell. After 4 h of incubation at 37 °C, cell supernatants were collected and stored at −80 °C for further analysis. A BD cytometric bead array human inflammatory cytokines kit was used according to manufacturer’s specification to quantify the concentrations of IL-8, IL-10, Il-6, Il-1 beta, TNF alpha and IL-12p70.

### 2.8. Antimicrobial Effect upon Storage

The stability of the OMVs’ antimicrobial effect was tested by maintaining the purified fractions at 4 °C for up to four weeks before testing them against *E. coli* DH5-alpha. After treating *E. coli* with different dilutions of OMVs for 18 h at 37 °C, the optical density was measured at 600 nm with a plate reader.

### 2.9. Bacteriomimetic Liposome Preparation

Bacteriomimetic liposomes were prepared by thin film hydration, as previously reported, with minor modifications [36]. A 6% (*w*/*v*) phospholipid solution was prepared by dissolving 1-hexadecanoyl-2-(9Z-octadecenoyl)-sn-glycero-3-phosphoethanolamine (POPE), 1-hexadecanoyl-2-(9Z-octadecenoyl)-sn-glycero-3-phospho-(1′-rac-glycerol) (sodium salt) (POPG) and 1,1′,2,2′-tetra-(9Z-octadecenoyl) cardiolipin (sodium salt) (CL) (Avanti Polar Lipids Inc., Alabaster, AL, USA) (weight ratio 70:20:10) in 5 mL of a chloroform-methanol blend (2:1) in a 250 mL round bottom flask. A Rotavapor R-205 (BÜCHI Labortechnik GmbH, Essen, Germany) was employed to remove the solvent under low pressure (60 min, 200 mbar, 135 rpm, 80 °C; 30 min, 40 mbar, 135 rpm, 80 °C). The remaining lipid film was rehydrated by adding 5 mL PBS (pH 7.4) containing 10% (*v*/*v*) ethanol and rotating for 60 min (70 °C, 135 rpm, atmospheric pressure). The obtained liposomes were sonicated for 60 min followed by 10 extrusion cycles at 70 °C, employing a Liposofast L-50 extruder (Avestin Europe GmbH, Mannheim, Germany).

### 2.10. Flow Cytometry and Confocal Laser Scanning Microscopy to Assess OMV Uptake into Mammalian Cells

Purified fractions of OMVs and a suspension of the bacteriomimetic liposome control were incubated with 2 µL of 1,1′-dioctadecyl-3,3,3′,3′-tetramethylindocarbocyanine perchlorate (DiI) (Molecular Probes Inc., Eugene, OR, USA) for 30 min at 37 °C. Afterwards, the suspensions were purified with a 10 mL column filled with Sepharose CL-2B (GE Life Science, UK) in PBS to remove any unbound dye. The fluorescence of the fractions was measured with a plate reader and the fractions with the highest concentrations were used. The DiI-stained particles were diluted 1:5 in cell culture medium with 1% penicillin and streptomycin (*v*/*v*) (Life Technologies Corporation, Grand Island, NY, USA) and incubated with cells at different time points.

For flow cytometry experiments, A549 and RAW 264.7 cells were seeded in 24-well plates with a density of 2 × 10^5^ cells per well and incubated for 48 h at 37 °C and 5% CO_2_.

Prior to flow cytometry (LSRFortessa, BD Bioscience, San Jose, CA, USA) analysis, the cells were washed with PBS and detached by using trypsin/EDTA (Life Technologies Limited, Paisley, UK) (A549) or a cell scraper (RAW 264.7). The cells were diluted with 2% (*v*/*v*) FCS in PBS and then added into FACS tubes for uptake analysis. Cells treated with PBS diluted in mammalian cell culture medium (1:5 dilution) were used as a negative control to set up the Phycoerythrin (PE) channel gate. A 10,000 live cells threshold was set to be analyzed from forward versus side scatter (FSC vs. SSC) gating. Single cells were determined by forward height versus forward area scatter (FCS-H vs. FCS-A) gating. The percentage of positive cells and the mean fluorescence intensity (MFI), which can be used to evaluate the amount of fluorescent particles taken up by the cells, were determined by analysis with the FlowJo 10.6.1 software (FlowJo LLC, Ashland, OR, USA) using the Phycoerythrin area channel (PE-A).

For confocal laser scanning microscopy (CLSM), the cells were seeded in 8-well chambers (SPL Life Sciences, Pocheon-si, Gyeonggi-do, Korea) with 4 × 10^4^ cells per well. After the incubation time points, the cells seeded in the 8-well chambers were washed with PBS and fixed with 3.7% (*v*/*v*) paraformaldehyde in PBS. The cells were stained with Alexa Fluor 488 Phalloidin (Life Technologies Coorporation, Eugene, OR, USA) for 1 h and DAPI (4′,6-Diamidino-2-phenylindole dihydrochloride) (Sigma-Aldrich Co., St. Louis, USA) for 30 min, washing the cells with PBS between the staining steps. The chambers were disassembled and mounted on a coverslip prior to CLSM analysis (Leica TCS SP8, Leica Microsystems, Wetzlar, Germany). The captured images were processed with the LAS X software (LAS X 1.8.013370, Leica Microsystems, Wetzlar, Germany).

### 2.11. Bacterial Culture

*Staphylococcus aureus* strain Newman and *Escherichia coli* strain DH5-alpha were obtained from the DSMZ (German Collection of Microorganisms and Cell Culture). Overnight cultures were prepared using 20 mL of brain heart infusion (BHI) broth (Becton; Dickinson and Company, Sparks, MD, USA) for *S. aureus* and Luria broth (LB) (Sigma-Aldrich; Co., St. Louis, MO, USA) for *E. coli* inoculated with a single colony. Cultures were incubated at 37 °C and 180 rpm.

### 2.12. Intracellular Infection

A549 cells were seeded (2 × 10^4^ cells/well) into 96-well plates and incubated for 48 h. Ten milliliters of the bacterial overnight culture was pelleted at 2000 × *g*, 4 °C, for 5 min and resuspended in PBS. The bacterial suspension in PBS was then diluted to approximately 2 × 10^6^ colony forming units per mL (CFU/mL) in mammalian cell medium. The cells were infected for 2 h (approximately multiplicity of infection (MOI) = 100), following washing with PBS and treatment with 50 µg/mL gentamicin for 30 min. After an additional washing step, cells were treated for 24 h with different concentrations of OMVs and controls, each diluted in cell culture medium. After 24 h incubation, the cells were lysed with HBSS (Hanks Buffered Saline Solution) (Life Technologies Limited, Paisley, UK) supplemented with 0.1% bovine serum albumin (Sigma-Aldrich Co., St. Louis, MO, USA) (*w*/*v*) and 0.1% Triton-X 100 (Sigma-Aldrich; Co., St. Louis, MO, USA) (*v*/*v*). Serial dilutions were prepared in a PBS solution containing 0.05% Tween-20 (Sigma-Aldrich, Co., St. Louis, MO, USA) (*v*/*v*) and inoculated on BHI agar plates (Becton; Dickinson and Company, Sparks, MD, USA), which were then incubated at 37 °C overnight. Afterwards, the single colonies were counted and the CFU/mL values calculated.

### 2.13. Statistical Analysis

The data is displayed as mean ± standard error of the mean (SEM). The number of independent experiments (*n*) is shown in each figure. The experiments and measurements were conducted at least in triplicates. The results were analyzed by GraphPad Prism 8.3 (GraphPad Software, San Diego, CA, USA), using Kruskal-Wallis test by ranks, followed by Dunn’s multiple comparisons test. Significant *P*-values were illustrated as * *p* < 0.05, ** *p* < 0.005, *** *p* < 0.0005 and **** *p* < 0.0001.

## 3. Results

### 3.1. OMVs are Successfully Isolated by Ultracentrifugation and SEC

**Figure 1:**
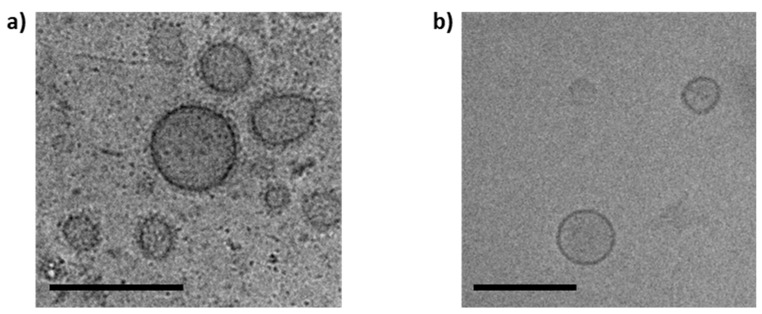
Cryo-TEM micrographs of (**a**) Cbfe23 outer membrane vesicles (OMVs) and (**b**) Cbv34 OMVs. Both OMVs are spherical and about 150 nm in size. Scale bars = 200 nm.

The OMVs were isolated from the myxobacterial cultures (Figure S1) and successfully separated from free proteins remaining in the pellet by SEC (Figure S2), using our standardized protocol [33]. The Cbfe23 OMVs were round-shaped and electron-dense, with a well delimited membrane, shown in the cryo-TEM micrographs (Figure 1). The main fractions of OMVs were about 120–150 nm in size (Figure S3). Cbfe23 OMVs had a zeta potential of −5.3 ± 0.7 mV while Cbv34 OMVs showed a zeta potential of −4.7 ± 0.6 mV, as recently reported by our group [33]. The liposomes used as a control for the uptake studies had a zeta potential of −24.5 ± 1.2 mV.

### 3.2. Myxobacterial OMVs Neither Affect Viability of Mammalian Cells nor Induce Cytotoxicity

**Figure 2:**
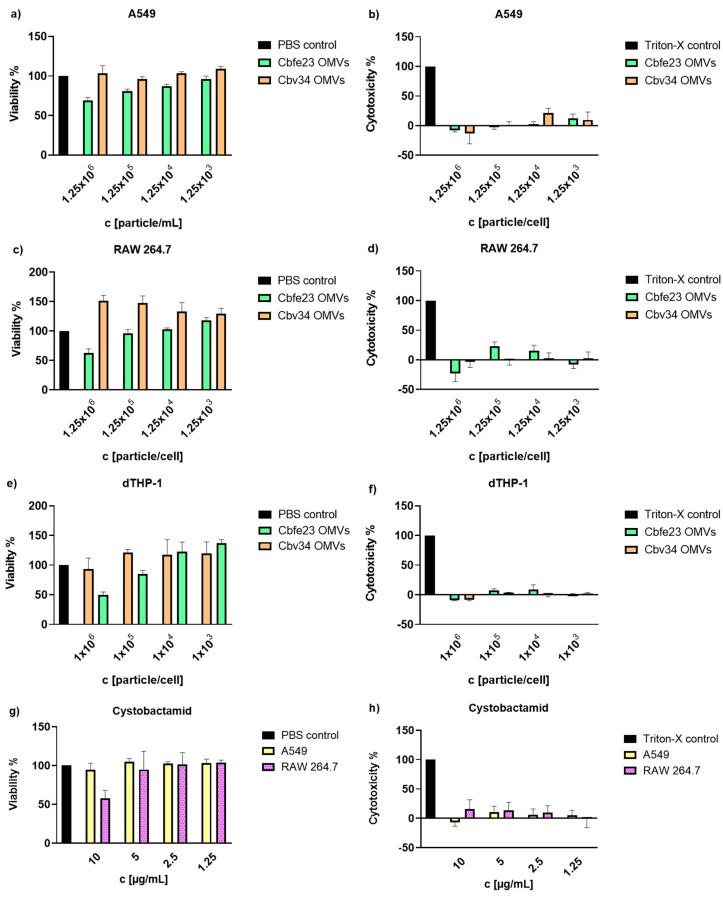
Cell viability and cytotoxicity of Cbv34 OMVs and Cbfe23 OMVs upon 24 h of treatment. Viability of (**a**) A549, (**c**) RAW 264.7 and (**e**) differentiated THP-1 cells, and lactate-dehydrogenase cytotoxicity of OMVs incubated with (**b**) A549, (**d**) RAW 264.7 and (**f**) differentiated THP-1 cells. Effect of cystobactamid on the (**g**) viability and (**h**) cytotoxicity of A549 and RAW 264.7 cells. Mean ± SEM, *n* = 3. No statistically significant differences were observed for any sample.

The viability of alveolar epithelial cells A549, the macrophage cell line RAW 264.7 and differentiated THP-1 were investigated upon 24 h of incubation with different OMV concentrations (Figure 2). Cbv34 OMVs did not impact the viability of the cells at any concentration tested. The Cbfe23 OMVs were well tolerated until concentrations of 125,000 OMVs/cell. Very high amounts of OMVs (i.e., 125,000 OMVs/cell) decreased the macrophage cell line viability by 50% (Figure 2c,e), and by around 60% when incubated with A549 cells (Figure 2a). A comparable effect was observed for serial dilutions of cystobactamid, the natural compound previously identified in Cbv34 OMVs (Figure 2g) [33]. Cbv34 OMVs increased viability above 100% when incubated with dTHP-1 cells. We had previously observed this effect, which may be due to the nutrient bolus of lipids provided by high amounts of OMVs [33]. To further evaluate this effect, the cytotoxicity was assessed by LDH assay. As seen in Figure 2b,d,f, we did not detect any underlying cytotoxicity in all concentrations of OMVs tested and a similar result was obtained from different concentrations of cystobactamid (Figure 2h).

### 3.3. Proinflammatory Cytokine Detection by Flow Cytometry

**Figure 3:**
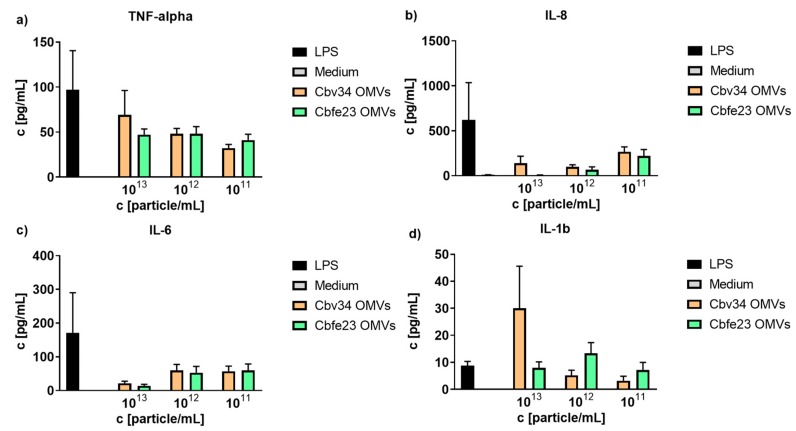
Cytokine detection of OMV-treated peripheral blood mononuclear cells (PBMCs). OMV-induced release of (**a**) TNF-alpha, (**b**) IL-8), (**c**) IL-6 and (**d**) IL 1-b at different concentrations. Mean ± SEM, *n* = 3.

To better understand the effect that OMVs may have on primary immune cells, we isolated peripheral blood mononuclear cells and incubated them with different concentrations of myxobacterial OMVs. As seen in Figure 3, in comparison to the positive control lipopolysaccharide, we only observed a tendency for the stimulation of release of TNF-alpha, IL-8, IL-6 and IL-1beta. Only at concentrations of 1 × 10^13^ particles/mL of Cbv34 OMVs, an increase in production of IL-1beta was found. The treatment with high concentrations of OMVs (1 × 10^13^ particles/mL) seemed to affect the viability of PBMCs, as seen by light microscopy (Figure S5).

### 3.4. Myxobacterial OMVs Are Able to Kill Planktonic S. aureus

**Figure 4:**
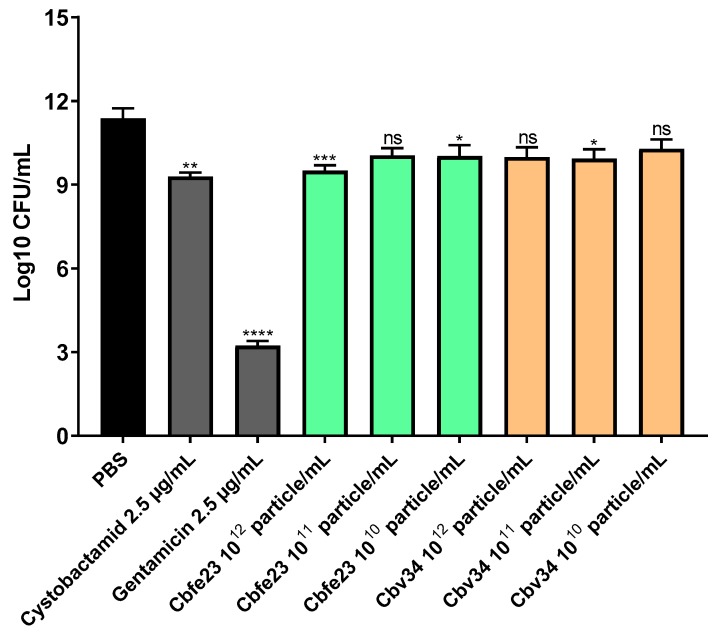
Antibacterial activity of Cbfe23 and Cbv34 OMVs against *S. aureus* strain Newman after 4 h of incubation at 37 °C. Mean ± SEM, *n* = 3. Significance was defined in comparison to the PBS control (black column) as * *p*-value < 0.05, *** *p* < 0.0005, **** *p*-value < 0.0001 and ns = not significant.

**Figure 5:**
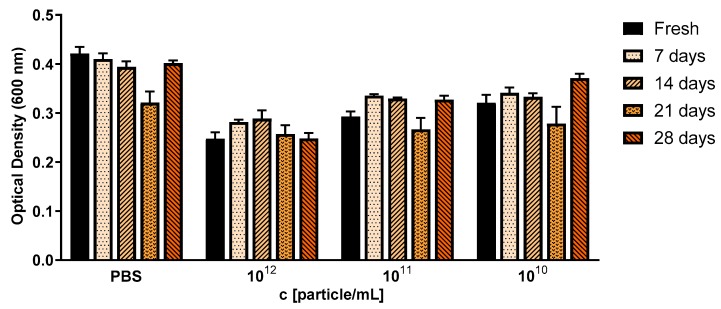
Antibacterial activity of Cbv34 OMVs against *E. coli* DH5-alpha right after isolation (fresh), and upon storage at 4 °C for 7, 14, 21 and 28 days. The treatment was done for 18 h at 37 °C. Mean ± SEM, *n* = 3.

OMVs had a killing effect at concentrations of approximately 1 × 10^12^ particles/mL. This correlates to a ratio of 3 × 10^3^ particles per CFU of *S. aureus* (Figure 4). We credit this effect to the cystobactamid compounds found in the OMV pellets (Figure S6). We were further interested to see if the antibacterial effect of myxobacterial OMVs is preserved during storage. For this, we tested whether Cbv34 OMVs are active against *E. coli* DH5-alpha after storage at 4 °C for up to 28 days based on our recent protocol [33]. Under these conditions, the antimicrobial activity of the OMVs remained potent and dose-dependent over time (Figure 5).

### 3.5. OMVs Are Taken Up by Mammalian Cells

**Figure 6:**
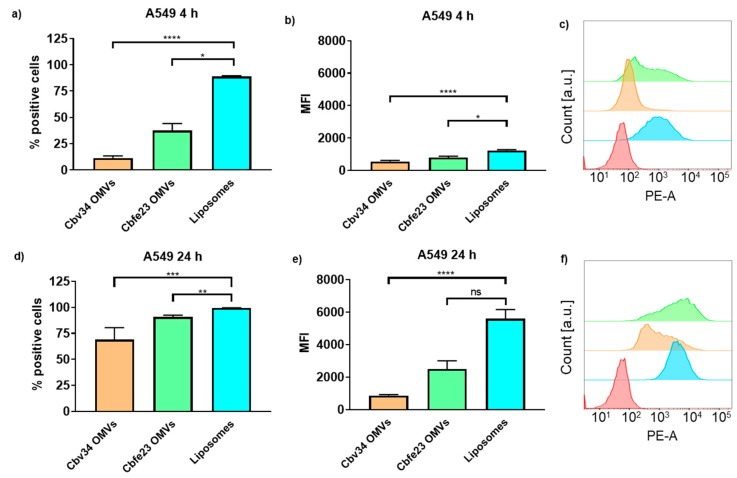
Interaction and uptake of OMVs and liposomes after incubation with A549 cells. The percentage of positive cells (which interacted with DiI-labelled OMVs) after (**a**) 4 h and (**d**) 24 h of incubation. The MFI after (**b**) 4 h and (**e**) 24 h of incubation. Representative histograms of Cbfe23 OMVs (green), Cbv34 OMVs (orange), liposomes (blue) and untreated control (red) after (**c**) 4 h and (**f**) 24 h of incubation. Mean ± SEM, *n* = 3. * *p* < 0.05, ** *p* < 0.005, *** *p* < 0.0005 and **** *p* < 0.0001.

**Figure 7:**
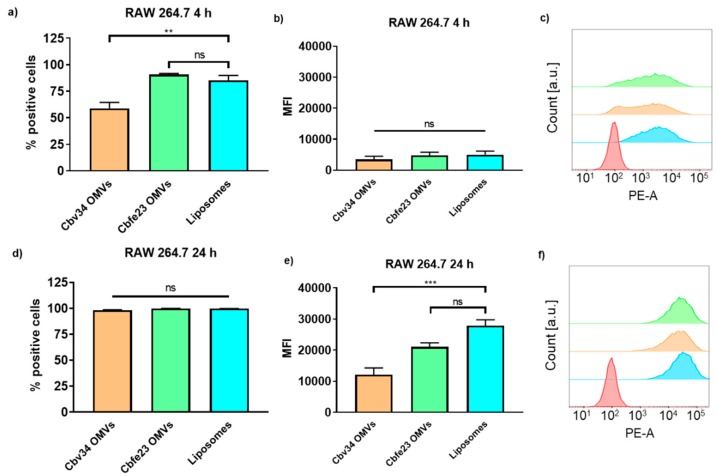
Interaction and uptake of OMVs and liposomes after incubation with RAW 264.7 cells. The percentage of positive cells (which interacted with DiI-labelled OMVs) after (**a**) 4 h and (**d**) 24 h of incubation. The MFI after (**b**) 4 h and (**e**) 24 h of incubation. Representative histograms of Cbfe23 OMVs (green), Cbv34 OMVs (orange), liposomes (blue) and untreated control (red) after (**c**) 4 h and (**f**) 24 h of incubation. Mean ± SEM, *n* = 3. ** *p* < 0.005, *** *p* < 0.0005 and ns = not significant.

**Figure 8:**
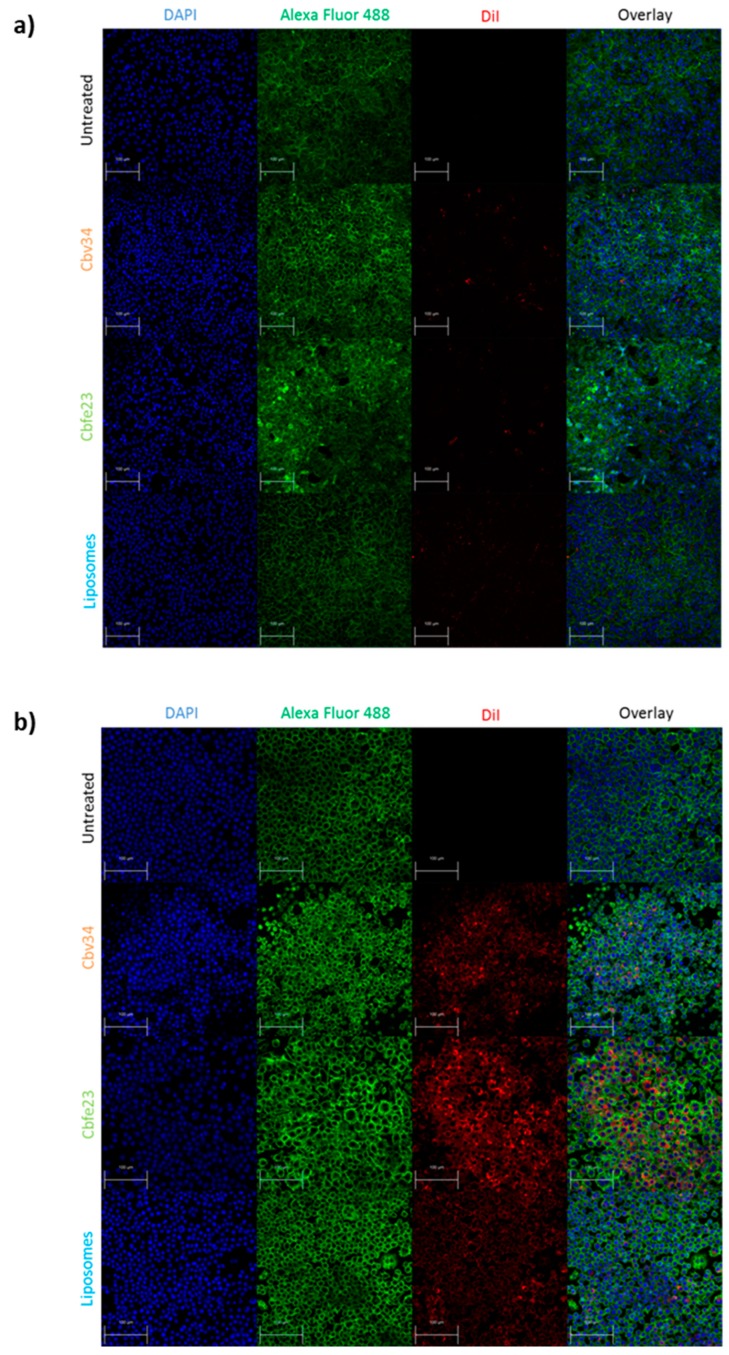
Uptake of fluorescent particles investigated by confocal laser scanning microscopy (CLSM) after 24 h of incubation with (**a**) A549 and (**b**) RAW 264.7 cells. The cells nuclei are stained with DAPI (blue), while the F-actin is labelled with Alexa Fluor 488 Phalloidin (green). OMVs and bacterial liposomes are stained with DiI (red). Scale bar = 100 µm.

The interaction with and uptake of the OMVs in A549 epithelial cells and RAW 264.7 immune cells were assessed by flow cytometry (Figure 6 and Figure 7) and CLSM (Figure 8). In comparison to liposomes prepared from bacterial phospholipids, we observed a significantly slower uptake of OMVs in A549 cells at 4 and 24 h of incubation (Figure 6a,d). The MFI of the liposomes was significantly higher when compared to the OMVs at the 4 h time point with A549 cells (Figure 6b). The MFI difference remained high between Cbv34 OMVs and liposomes at 24 h, but it was not significant when Cbfe23 OMVs and liposomes were compared (Figure 6e). As for the uptake into RAW 264.7 cells, Cbfe23 OMVs and bacterial liposomes did not show any significant difference in terms of percentage of positive cells and MFI (Figure 7), both having a percentage of positive cells of nearly 90% at 4 h incubation time (Figure 7a). However, Cbv34 OMVs had a significantly lower percentage of positive cells when compared to the bacterial liposomes at 4 h (Figure 7a) and lower MFI at 24 h (Figure 7e). At the 24 h time point, all particles led to 100% of fluorescent cells with RAW 264.7 cells (Figure 7d). MFI remained comparable between all the samples at the 4 h time point (Figure 7b). The representative histograms of the uptake of DiI-stained particles in A549 cells (Figure 6c,f) and RAW 264.7 cells (Figure 7c,f) show the shift in the histograms of the samples compared to the untreated control (red).

As for the localization of the particles within the cells, we observed little fluorescence signal coming from the particles after 4 h of incubation with A549 (Figure S7), and a higher fluorescence of Cbfe23 OMVs when incubated with RAW 264.7 cells for 4 h (Figure S8), when compared to the Cbv34 OMVs. Even though we saw DiI-stained Cbv34 and Cbfe23 OMVs within the cells, we did not observe colocalization with the cytoskeleton at any investigated time point (Figure 8). We noticed a removal of OMVs by PBS washing during the staining process, which may indicate that they are mostly bound onto the cell surface. When the cells were washed once with PBS and fixed, we observed a high number of fluorescent particles in A549 cells (Figures S9 and S10).

### 3.6. OMVs Are Able to Kill Intracellular Pathogens

**Figure 9:**
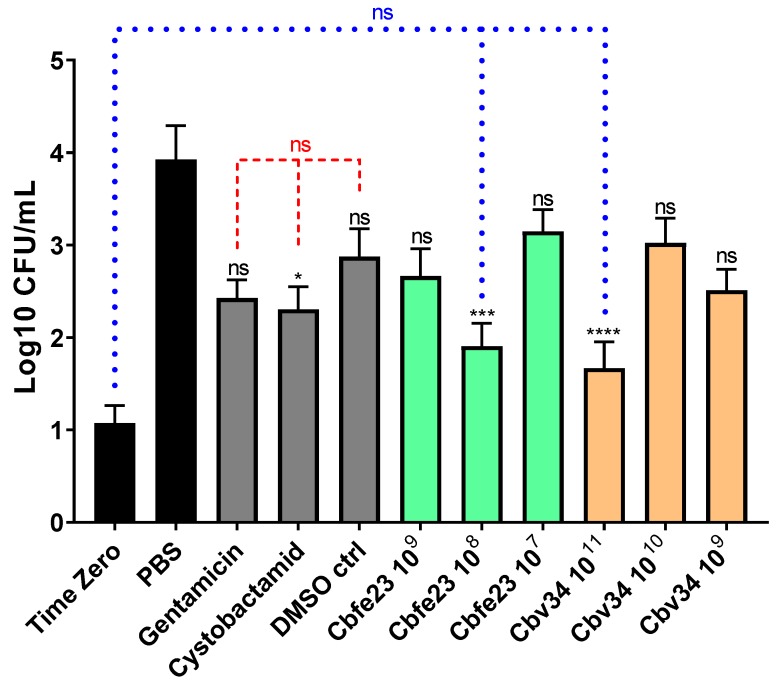
Antibacterial effect of OMVs against intracellular *S. aureus* after 24 h of treatment. Mean ± SEM, *n* = 3–4 independent experiments. Significance was defined in relation to the PBS control as ns = not significant, * *p*-value < 0.05, *** *p*-value < 0.0005 and **** *p*-value < 0.0001. Red dashed lines represent the comparison between gentamicin 10 µg/mL, cystobactamid 10 µg/mL and the DMSO control = not significant. Blue dotted lines represent the comparison between the time zero (2 h bacterial uptake), Cbfe23 10^8^ particles/mL and Cbv34 10^11^ particles/mL.

We investigated the ability of myxobacterial OMVs to treat intracellular infections caused by *S. aureus* in A549 cells. The most active concentrations were 10^8^ OMVs/mL and 10^11^ OMVs/mL for Cbfe23 and Cbv34 OMVs, respectively (Figure 9). The highest concentrations of Cbfe23 OMVs were not tested, because they negatively influenced the viability of the cells, as shown in Figure 2a. Gentamicin is soluble in water, while cystobactamid is dissolved in DMSO (dimethyl sulfoxide), so we included DMSO as a solvent control. The DMSO might contribute to the antibacterial effect of the free cystobactamid, as we observed a tendency of lower CFU amounts in the DMSO sample alone. Both free gentamicin and cystobactamid, together with the solvent control DMSO, were not significantly different when compared among themselves. Our OMVs, which are in aqueous suspension, showed a more pronounced reduction in bacterial growth compared to free antibiotics. By comparing this with the number of bacteria present in the cells after 2 h of uptake (time zero), we see that the effect of the OMVs against *S. aureus* is bacteriostatic. This effect was also confirmed by the statistical analysis, which did not show a significant difference in bacterial growth between the most effective OMV concentrations and the time zero control (blue dashed line in Figure 9).

## 4. Discussion

Cbv34 and Cbfe23 OMVs were successfully isolated by differential centrifugation and SEC, presenting a size of approximately 120–150 nm, which is an advantageous size for interaction with target cells and accumulation in inflamed infectious tissue [37]. The Cryo-TEM images showed spherical forms for Cbv34 OMVs, but for Cbfe23 OMVs, rod-shaped particles were also seen (Figure S4). We suspect that these particles might be fragments of outer membrane tubes (OMTs), which are known to be produced by some myxobacterial strains [38].

As for the viability and cytotoxicity, we showed low to absent toxicity for Cbv34 OMVs, as also reported in literature [33]. Cbfe23 OMVs, which were studied here for the first time, reduced the viability of the cells only at a concentration of 125,000 OMVs/cell. This might be caused by a higher load of antibiotic in comparison to Cbv34 OMVs, since we could see that a concentration of 10 µg/mL cystobactamid also had a similar negative effect on cell viability. No dramatic cytotoxicity was detected at any concentrations tested for either OMV. We have tested whether our OMVs induce the production of proinflammatory cytokines to better understand the potential immunogenicity of our carriers. IL-6, which is a multifunctional cytokine, and TNF, are released in response to inflammation and mediate reactions to its effects, such as fever [39,40]. IL-8 and IL-1b induce chemotaxis and play a key role in inflammation, recruiting neutrophils to the site of infection [41,42,43]. Our OMVs promoted a mild release of proinflammatory cytokines compared to the LPS control. As an exception, the concentration of 10^13^ Cbfe23 OMVs/mL stimulated a high production of IL-1beta, but possibly induced cell death, as observed by light microscopy (Figure S5). IL-1beta is known to be negatively influenced in production by the use of molecules that induce autophagy [44], which contributes to cell survival. This result might also lead to a clue about the mechanisms of how Cbfe23 OMVs induce cell death at high concentrations.

Even though high concentrations of OMVs were needed to inhibit the growth of planktonic bacteria, concentrations of 10^12^ OMVs/mL and higher did not seem to be required for an antibacterial effect against intracellular pathogens. When intracellular CFUs were assessed to investigate the OMVs’ activity against *S. aureus*, a bacterial growth inhibition was identified, especially from Cbfe23 OMVs. In literature, the Cbfe23 strain has not been reported to produce cystobactamids [45]. Here, we modified the composition of the bacterial culture medium by using phytone as a carbon source, instead of the previously reported skimmed milk and soy flour [45]. Due to this modification, we were able to identify cystobactamids 919-1 and 920-1 [46,47,48] as the active compounds in the OMV extract (Figure S6). For the potential application of OMVs as an antibacterial therapy in the clinic, their formulations must be stable for a certain period, preferably without any special storage conditions, such as deep freezing at −80 °C and the use of cryoprotectants. Since we have already showed an antibacterial effect of Cbv34 OMVs against *E. coli* [26] and the easy handling of this bacterium, we investigated OMV storage stability at 4 °C with this model pathogen. We found that the dose-dependent antibacterial activity of Cbv34 OMVs was conserved during 28 days of storage. This result matches recent findings that show extracellular vesicles’ inherent stability under different storage conditions [49,50].

Compared to bacteriomimetic liposomes, OMVs showed different uptake kinetics with immune and epithelial cells. While liposomes are rapidly taken up in all cell types at high amounts, OMVs seemed to have a preferentially better uptake in immune cells than in epithelial cells. Surface charge may play a role in this mechanism, but surface proteins of OMVs are also important for this effect, as was shown for mammalian vesicles [51]. As we show, OMVs have an antibacterial effect against internalized *S. aureus* in A549 cells. Both Cbfe23 and Cbv34 OMVs showed a higher antibacterial effect, when compared to the untreated control, than the free gentamicin and cystobactamid. Free antibiotics generally have a low permeation through mammalian cell membranes and thus favor intracellular infections, which may become persistent and difficult to treat. The use of myxobacterial OMVs as nanocarriers could possibly overcome the cellular barrier and successfully deliver the antibacterial compound to the lungs, potentially being administered locally as an aerosol.

The next steps to further evaluate myxobacterial OMVs as drug carriers are to rule out their immunogenicity in complex in vitro models of inflammation [52], and the evaluation of their antibacterial activity in an infected animal model.

## 5. Conclusions

OMVs from Cbv34 and Cbfe23 are not only low in toxicity in different cell lines, but also in primary immune cells. They are able to mediate killing of one of the most important Gram-positive problem pathogens, *S. aureus*. These OMVs are able to be taken up into infected cells and showed efficient bacteriostatic effect against intracellular *S. aureus* infections.

## Supplementary Materials

The following are available online at https://www.mdpi.com/2073-4409/9/1/194/s1, Figure S1: Morphology of Cbfe23 (passage 3, 5 days in culture) and Cbv34 (passage 9, 7 days in culture), Figure S2: Particle size measured by dynamic light scattering and protein concentration of Cbfe23 size exclusion chromatography fractions (1 mL each) were measured by BCA (bicinchoninic acid) assay (Sigma-Aldrich Co., St. Louis, MO, USA), Figure S3: Representative graphs of size distributions measured by nanoparticle tracking analysis of a) Cbv34 and b) Cbfe23 OMVs, Figure S4: Cryo-TEM micrographs of Cbfe23 pellets after ultracentrifugation, Figure S5: Light microscopy images showing the morphology of peripheral blood mononuclear cells (PBMCs) after 4 h treatment with OMVs and controls, Figure S6: a) LC-MS Base peak chromatogram of the OMV extract (black) highlighting the two major cystobactamids: cystobactamid 919-1 (A, red, m/z 920.31 ± 0.05 Da) and cystobactamid 920-1 (B, blue, m/z 921.30 ± 0.05 Da) as extracted ion chromatograms, Figure S7: Uptake/interaction of OMVs and liposomes with A549 cells after 4 h incubation measured by confocal laser scanning microscopy, Figure S8: Uptake/interaction of OMVs and liposomes with RAW 264.7 cells after 4 h of incubation measured by confocal laser scanning microscopy, Figure S9: Uptake/interaction imaging of OMVs and liposomes after 4 h incubation with A549, following one single wash with PBS and fixation by confocal laser scanning microscopy, Figure S10: Uptake/interaction imaging of OMVs and liposomes after 24 h incubation with A549, following one single wash with PBS and fixation by confocal laser scanning microscopy.

## References

[1] Troeger C., Blacker B., Khalil I.A., Rao P.C., Cao J., Zimsen S.R.M., Albertson S.B., Deshpande A., Farag T., Abebe Z. (2018). Estimates of the global, regional, and national morbidity, mortality, and aetiologies of lower respiratory infections in 195 countries, 1990–2016: A systematic analysis for the Global Burden of Disease Study 2016. Lancet Infect. Dis..

[2] Naghavi M., Abajobir A.A., Abbafati C., Abbas K.M., Abd-Allah F., Abera S.F., Aboyans V., Adetokunboh O., Ärnlöv J., Afshin A. (2017). Global, regional, and national age-sex specific mortality for 264 causes of death, 1980–2016: A systematic analysis for the Global Burden of Disease Study 2016. Lancet.

[3] Sherrard L.J., Tunney M.M., Elborn J.S. (2014). Infections in chronic lung diseases 2 Antimicrobial resistance in the respiratory microbiota of people with cystic fi brosis. Lancet.

[4] Lommatzsch S.T., Aris R. (2009). Genetics of cystic fibrosis. Semin. Respir. Crit. Care Med..

[5] Ahmed M.I., Mukherjee S. (2018). Treatment for chronic methicillin-sensitive Staphylococcus aureus pulmonary infection in people with cystic fibrosis. Cochrane Database Syst. Rev..

[6] Ulrich M., Herbert S., Berger J., Bellon G., Louis D., Münker G., Döring G. (1998). Localization of Staphylococcus aureus in Infected Airways of Patients with Cystic Fibrosis and in a Cell Culture Model of S. aureus Adherence. Am. J. Respir. Cell Mol. Biol..

[7] Tranchemontagne Z.R., Camire R.B., O’Donnell V.J., Baugh J., Burkholder K.M. (2016). Staphylococcus aureus Strain USA300 Perturbs Acquisition of Lysosomal Enzymes and Requires Phagosomal Acidification for Survival inside Macrophages. Infect. Immun..

[8] Flannagan R.S., Heit B., Heinrichs D.E. (2016). Intracellular replication of Staphylococcus aureus in mature phagolysosomes in macrophages precedes host cell death, and bacterial escape and dissemination. Cell. Microbiol..

[9] Fraunholz M., Sinha B. (2012). Intracellular staphylococcus aureus: Live-in and let die. Front. Cell. Infect. Microbiol..

[10] Löffler B., Tuchscherr L., Niemann S., Peters G. (2014). Staphylococcus aureus persistence in non-professional phagocytes. Int. J. Med. Microbiol..

[11] Barcia-Macay M., Seral C., Mingeot-Leclercq M.-P., Tulkens P.M., Van Bambeke F. (2006). Pharmacodynamic evaluation of the intracellular activities of antibiotics against Staphylococcus aureus in a model of THP-1 macrophages. Antimicrob. Agents Chemother..

[12] Vaudaux P., Waldvogel F.A. (1979). Gentamicin antibacterial activity in the presence of human polymorphonuclear leukocytes. Antimicrob. Agents Chemother..

[13] Carlier M.-B., Zenebergh A., Tulkens P.M. (1987). Cellular uptake and subcellular distribution of roxithromycin and erythromycin in phagocytic cells. J. Antimicrob. Chemother..

[14] Jacobs R.F., Wilson C.B. (1983). Activity of Antibiotics in Chronic Granulomatous Disease Leukocytes. Pediatr. Res..

[15] Jacobs R.F., Wilson C.B. (1983). Intracellular penetration and antimicrobial activity of antibiotics. J. Antimicrob. Chemother..

[16] Bongers S., Hellebrekers P., Leenen L.P.H., Koenderman L., Hietbrink F. (2019). Intracellular Penetration and Effects of Antibiotics on Staphylococcus aureus Inside Human Neutrophils: A Comprehensive Review. Antibiotics.

[17] Anversa Dimer F., de Souza Carvalho-Wodarz C., Goes A., Cirnski K., Herrmann J., Schmitt V., Pätzold L., Abed N., De Rossi C., Bischoff M. (2020). PLGA nanocapsules improve the delivery of clarithromycin to kill intracellular Staphylococcus aureus and Mycobacterium abscessus. Nanomed. Nanotechnol. Biol. Med..

[18] Menina S., Eisenbeis J., Kamal M.A.M., Koch M., Bischoff M., Gordon S., Loretz B., Lehr C. (2019). Bioinspired Liposomes for Oral Delivery of Colistin to Combat Intracellular Infections by *Salmonella enterica*. Adv. Healthc. Mater..

[19] Castoldi A., Empting M., De Rossi C., Mayr K., Dersch P., Hartmann R., Müller R., Gordon S., Lehr C.M. (2019). Aspherical and Spherical InvA497-Functionalized Nanocarriers for Intracellular Delivery of Anti-Infective Agents. Pharm. Res..

[20] Yang X., Shi G., Guo J., Wang C., He Y. (2018). Exosome-encapsulated antibiotic against intracellular infections of methicillin-resistant Staphylococcus aureus. Int. J. Nanomed..

[21] Goes A., Fuhrmann G. (2018). Biogenic and Biomimetic Carriers as Versatile Transporters to Treat Infections. ACS Infect. Dis..

[22] Forier K., Raemdonck K., De Smedt S.C., Demeester J., Coenye T., Braeckmans K. (2014). Lipid and polymer nanoparticles for drug delivery to bacterial biofilms. J. Control. Release.

[23] Reichenbach H. (1999). The ecology of the myxobacteria. Environ. Microbiol..

[24] Wu Y., Jiang Y., Kaiser D., Alber M. (2007). Social interactions in myxobacterial swarming. PLoS Comput. Biol..

[25] Reichenbach H., Gerth K., Irschik H., Kunze B., Höfle G. (1988). Myxobacteria: A source of new antibiotics. Trends Biotechnol..

[26] Weissman K.J., Müller R. (2010). Myxobacterial secondary metabolites: Bioactivities and modes-of-action. Nat. Prod. Rep..

[27] Reichenbach H. (2001). Myxobacteria, producers of novel bioactive substances. J. Ind. Microbiol. Biotechnol..

[28] Hoffmann T., Krug D., Bozkurt N., Duddela S., Jansen R., Garcia R., Gerth K., Steinmetz H., Müller R. (2018). Correlating chemical diversity with taxonomic distance for discovery of natural products in myxobacteria. Nat. Commun..

[29] Schwechheimer C., Kuehn M.J. (2015). Outer-membrane vesicles from Gram-negative bacteria: Biogenesis and functions. Nat. Rev. Microbiol..

[30] Kulp A., Kuehn M.J. (2010). Biological Functions and Biogenesis of Secreted Bacterial Outer Membrane Vesicles. Annu. Rev. Microbiol..

[31] Woith E., Fuhrmann G., Melzig M.F. (2019). Extracellular Vesicles—Connecting Kingdoms. Int. J. Mol. Sci..

[32] Evans A.G.L., Davey H.M., Cookson A., Currinn H., Cooke-Fox G., Stanczyk P.J., Whitworth D.E. (2012). Predatory activity of Myxococcus xanthus outer-membrane vesicles and properties of their hydrolase cargo. Microbiology.

[33] Schulz E., Goes A., Garcia R., Panter F., Koch M., Müller R., Fuhrmann K., Fuhrmann G. (2018). Biocompatible bacteria-derived vesicles show inherent antimicrobial activity. J. Control. Release.

[34] Baumann S., Herrmann J., Raju R., Steinmetz H., Mohr K.I., Hüttel S., Harmrolfs K., Stadler M., Müller R. (2014). Cystobactamids: Myxobacterial Topoisomerase Inhibitors Exhibiting Potent Antibacterial Activity. Angew. Chem. Int. Ed..

[35] Wang M., Carver J.J., Phelan V.V., Sanchez L.M., Garg N., Peng Y., Nguyen D.D., Watrous J., Kapono C.A., Luzzatto-Knaan T. (2016). Sharing and community curation of mass spectrometry data with Global Natural Products Social Molecular Networking. Nat. Biotechnol..

[36] Graef F., Vukosavljevic B., Michel J.-P., Wirth M., Ries O., De Rossi C., Windbergs M., Rosilio V., Ducho C., Gordon S. (2016). The bacterial cell envelope as delimiter of anti-infective bioavailability – An in vitro permeation model of the Gram-negative bacterial inner membrane. J. Control. Release.

[37] He C., Hu Y., Yin L., Tang C., Yin C. (2010). Effects of particle size and surface charge on cellular uptake and biodistribution of polymeric nanoparticles. Biomaterials.

[38] Wei X., Vassallo C.N., Pathak D.T., Wall D. (2014). Myxobacteria Produce Outer Membrane-Enclosed Tubes in Unstructured Environments. J. Bacteriol..

[39] Banks W.A., Kastin A.J., Gutierrez E.G. (1994). Penetration of interleukin-6 across the murine blood-brain barrier. Neurosci. Lett..

[40] Coceani F., Lees J., Mancilla J., Belizario J., Dinarello C.A. (1993). Interleukin-6 and tumor necrosis factor in cerebrospinal fluid: Changes during pyrogen fever. Brain Res..

[41] Harada A., Sekido N., Akahoshi T., Wada T., Mukaida N., Matsushima K. (1994). Essential involvement of interleukin-8 (IL-8) in acute inflammation. J. Leukoc. Biol..

[42] Baggiolini M., Loetscher P., Moser B. (1995). Interleukin-8 and the chemokine family. Int. J. Immunopharmacol..

[43] Sauder D.N., Mounessa N.L., Katz S.I., Dinarello C.A., Gallin J.I. (1984). Chemotactic cytokines: The role of leukocytic pyrogen and epidermal cell thymocyte-activating factor in neutrophil chemotaxis. J. Immunol..

[44] Shaw S.Y., Tran K., Castoreno A.B., Peloquin J.M., Lassen K.G., Khor B., Aldrich L.N., Tan P.H., Graham D.B., Kuballa P. (2013). Selective modulation of autophagy, innate immunity, and adaptive immunity by small molecules. ACS Chem. Biol..

[45] Raju R., Mohr K.I., Bernecker S., Herrmann J., Müller R. (2015). Cystodienoic acid: A new diterpene isolated from the myxobacterium Cystobacter sp.. J. Antibiot..

[46] Baumann S., Herrmann J., Raju R., Steinmetz H., Mohr K.I. (2014). Cystobactamids: Myxobacterial topoisomerase inhibitors exhibiting broad spectrum antibacterial activity. Angew. Chem..

[47] Planke T., Moreno M., Hüttel S., Fohrer J., Gille F., Norris M.D., Siebke M., Wang L., Müller R., Kirschning A. (2019). Cystobactamids 920-1 and 920-2: Assignment of the Constitution and Relative Configuration by Total Synthesis. Org. Lett..

[48] Cheng B., Müller R., Trauner D. (2017). Total Syntheses of Cystobactamids and Structural Confirmation of Cystobactamid 919-2. Angew. Chem. Int. Ed..

[49] Frank J., Richter M., de Rossi C., Lehr C.-M., Fuhrmann K., Fuhrmann G. (2018). Extracellular vesicles protect glucuronidase model enzymes during freeze-drying. Sci. Rep..

[50] Schulz E., Karagianni A., Koch M., Fuhrmann G. (2019). Hot EVs – how temperature affects extracellular vesicles. Eur. J. Pharm. Biopharm..

[51] Saari H., Lázaro-Ibáñez E., Viitala T., Vuorimaa-Laukkanen E., Siljander P., Yliperttula M. (2015). Microvesicle- and exosome-mediated drug delivery enhances the cytotoxicity of Paclitaxel in autologous prostate cancer cells. J. Control. Release.

[52] Susewind J., de Souza Carvalho-Wodarz C., Repnik U., Collnot E.-M., Schneider-Daum N., Griffiths G.W., Lehr C.-M. (2015). A 3D co-culture of three human cell lines to model the inflamed intestinal mucosa for safety testing of nanomaterials. Nanotoxicology.

