# Supplementary material for: Myxobacteria-Derived Outer Membrane Vesicles: Potential Applicability Against Intracellular Infections
Source: Cells. 2020 Jan 12;9(1):194. doi: 10.3390/cells9010194 (PMC7017139; doi:10.3390/cells9010194)
Supplement: Supplementary file 1 [file cells-09-00194-s001.zip › cells-675241-SI.docx]

Supplementary Material

Myxobacteria-derived outer membrane vesicles: potential applicability against intracellular infections

Adriely Goes, Philipp Lapuhs, Thomas Kuhn, Eilien Schulz, Robert Richter, Fabian Panter, Charlotte Dahlem, Marcus Koch, Ronald Garcia, Alexandra K. Kiemer, Rolf Müller and Gregor Fuhrmann


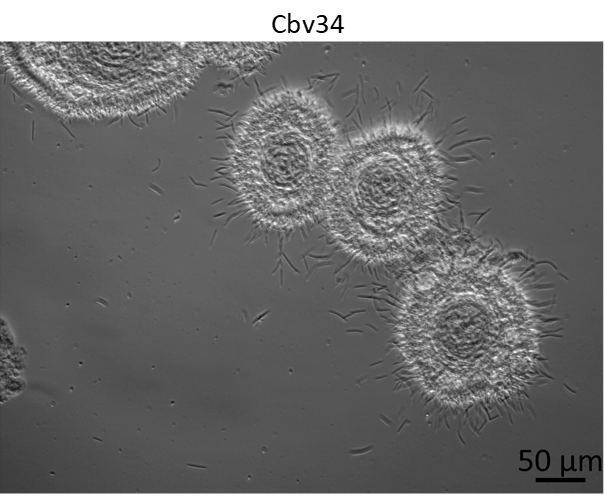

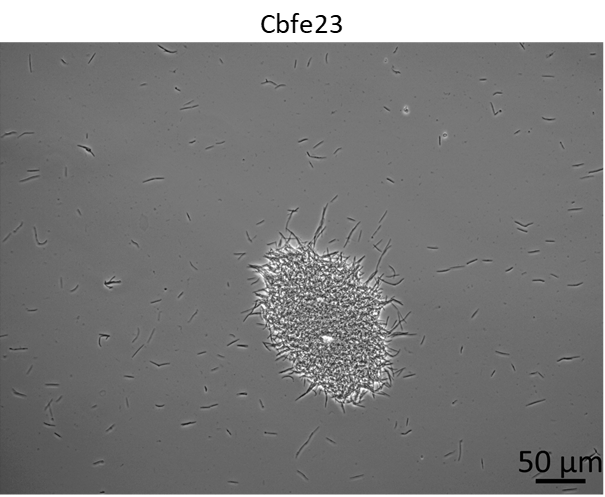


**Figure S1:** Morphology of Cbfe23 (passage 3, 5 days in culture) and Cbv34 (passage 9, 7 days in culture). Myxobacteria form cell aggregates in liquid culture. Images were taken with a light microscope (Zeiss, Germany). Scale bars = 50 µm.

**Figure S2:** Particle size measured by dynamic light scattering and protein concentration of Cbfe23 size exclusion chromatography fractions (1 mL each) were measured by BCA (bicinchoninic acid) assay (Sigma-Aldrich Co., St. Louis, MO, USA). Mean ± SEM, *n* = 3.


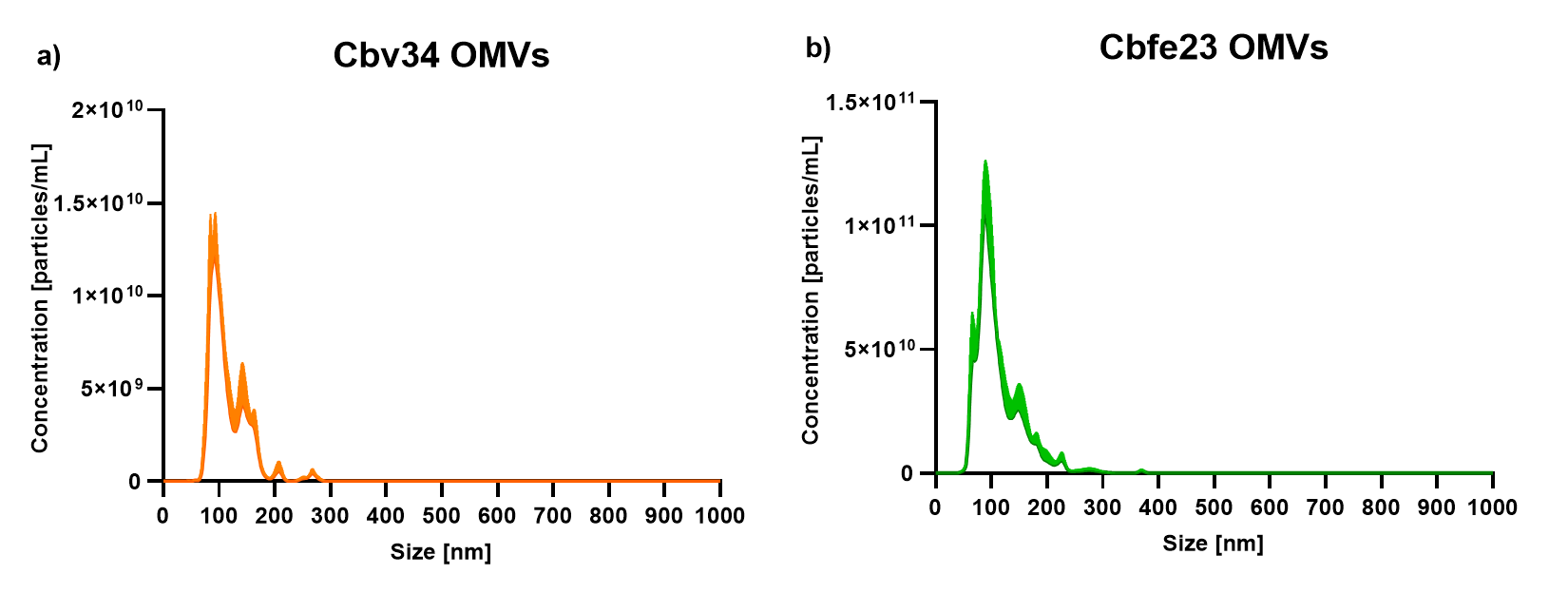


**Figure S3:** Representative graphs of size distributions measured by nanoparticle tracking analysis of a) Cbv34 and b) Cbfe23 OMVs. Mean + SEM.


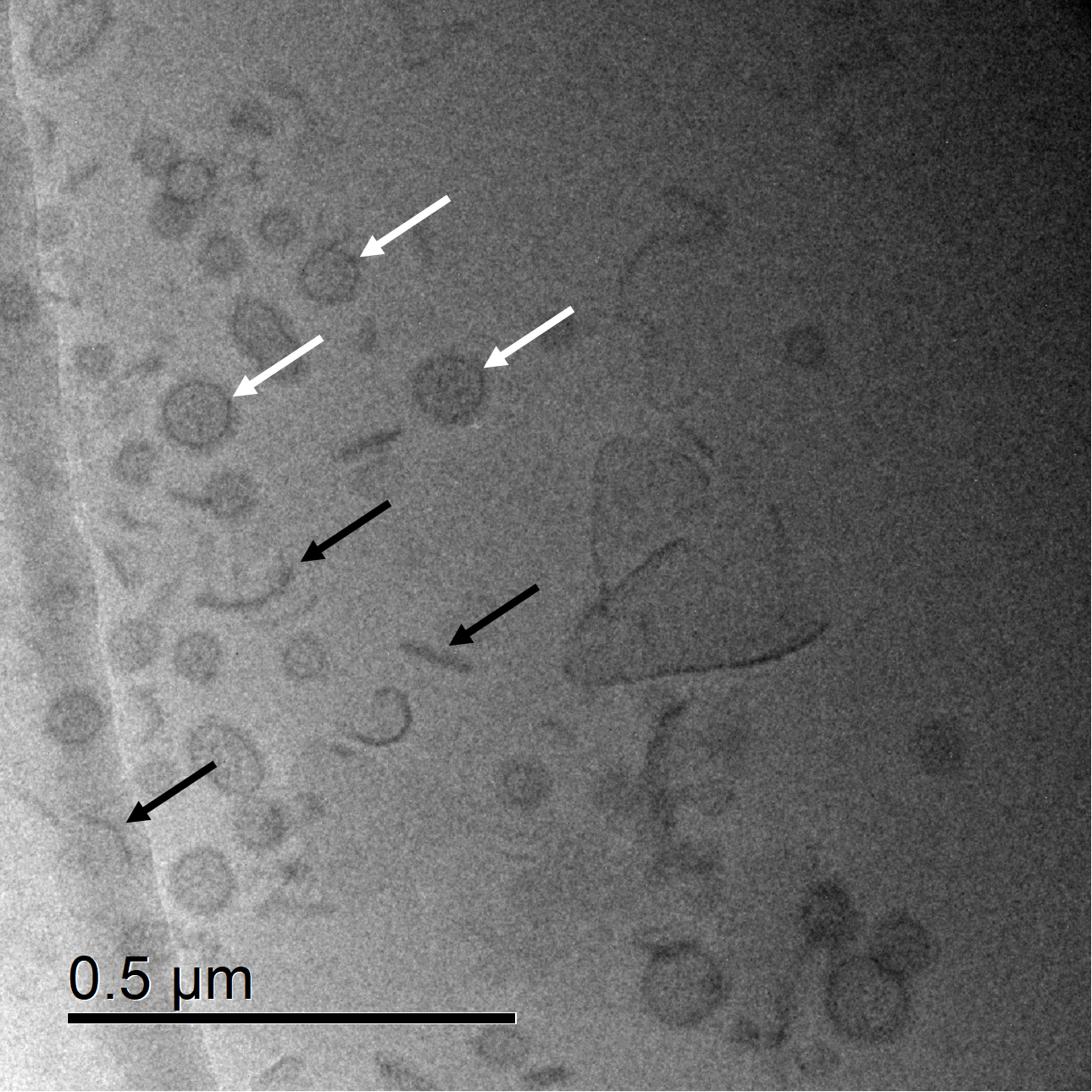

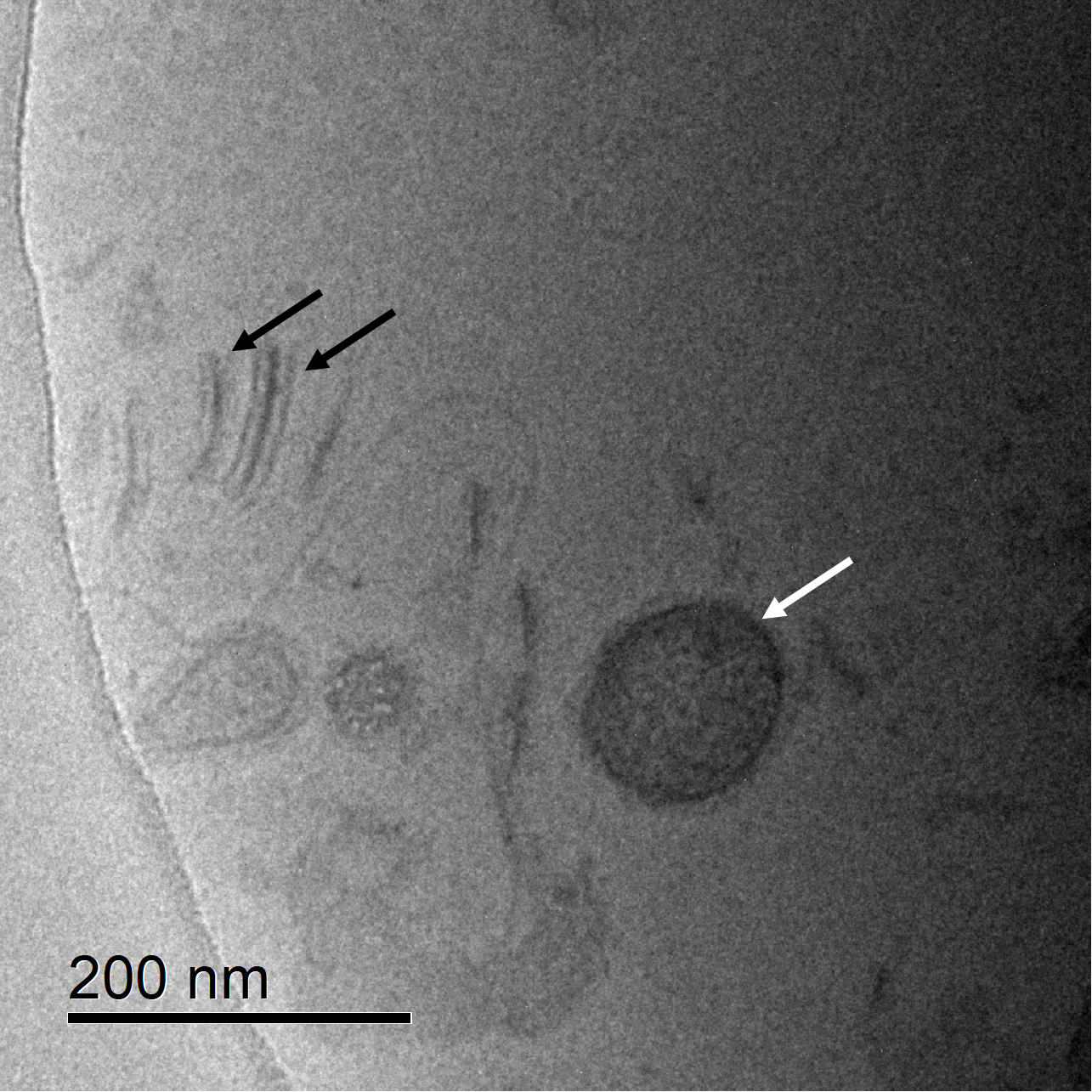


**a)**

**b)**

**Figure S4:** Cryo-TEM micrographs of Cbfe23 pellets after ultracentrifugation. The white arrows indicate OMVs with spherical shape and electron dense cores. The black arrows indicate rod-shaped electron dense particles of similar size as the OMVs.


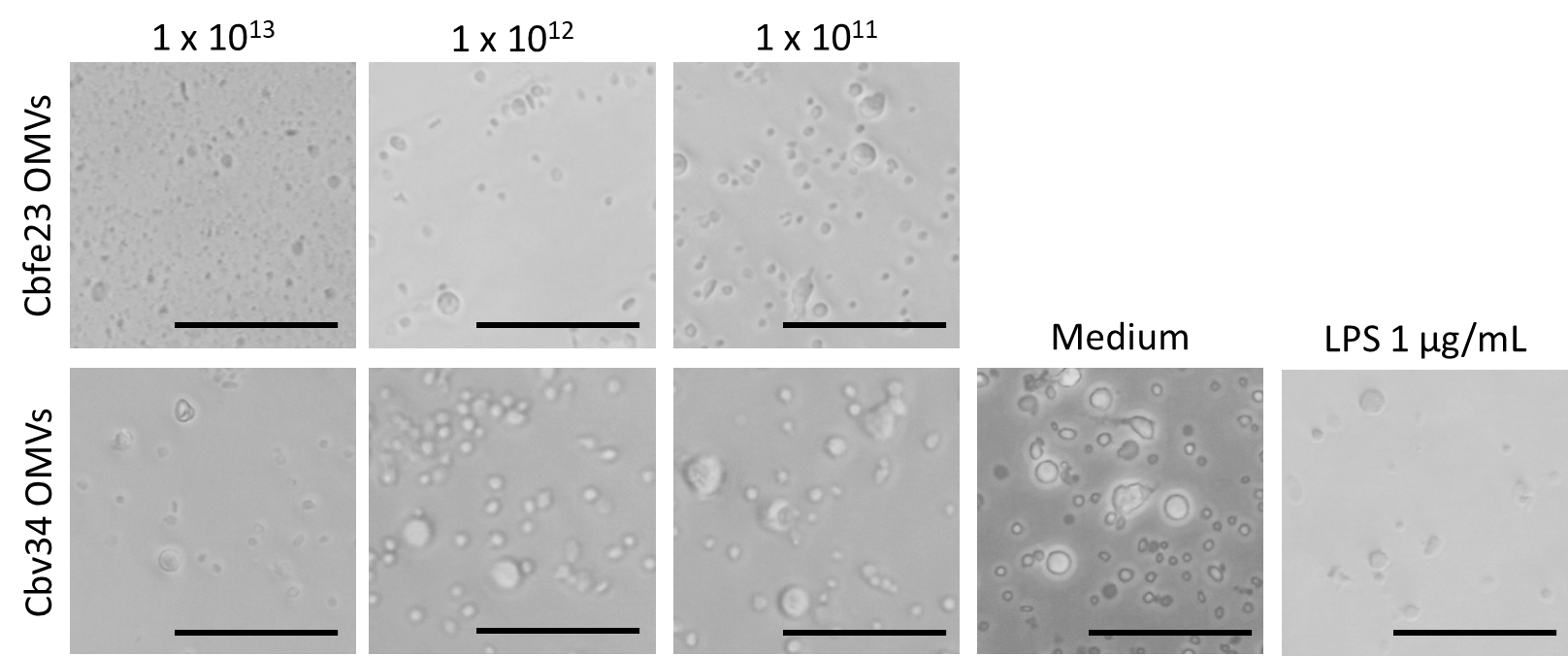


**Figure S5:** Light microscopy images showing the morphology of peripheral blood mononuclear cells (PBMCs) after 4 h treatment with OMVs and controls. The viability of the cells appeared to be compromised upon treatment with high concentrations of OMVs (1×10^13^ OMVs/mL). Scale bars = 50 µm.


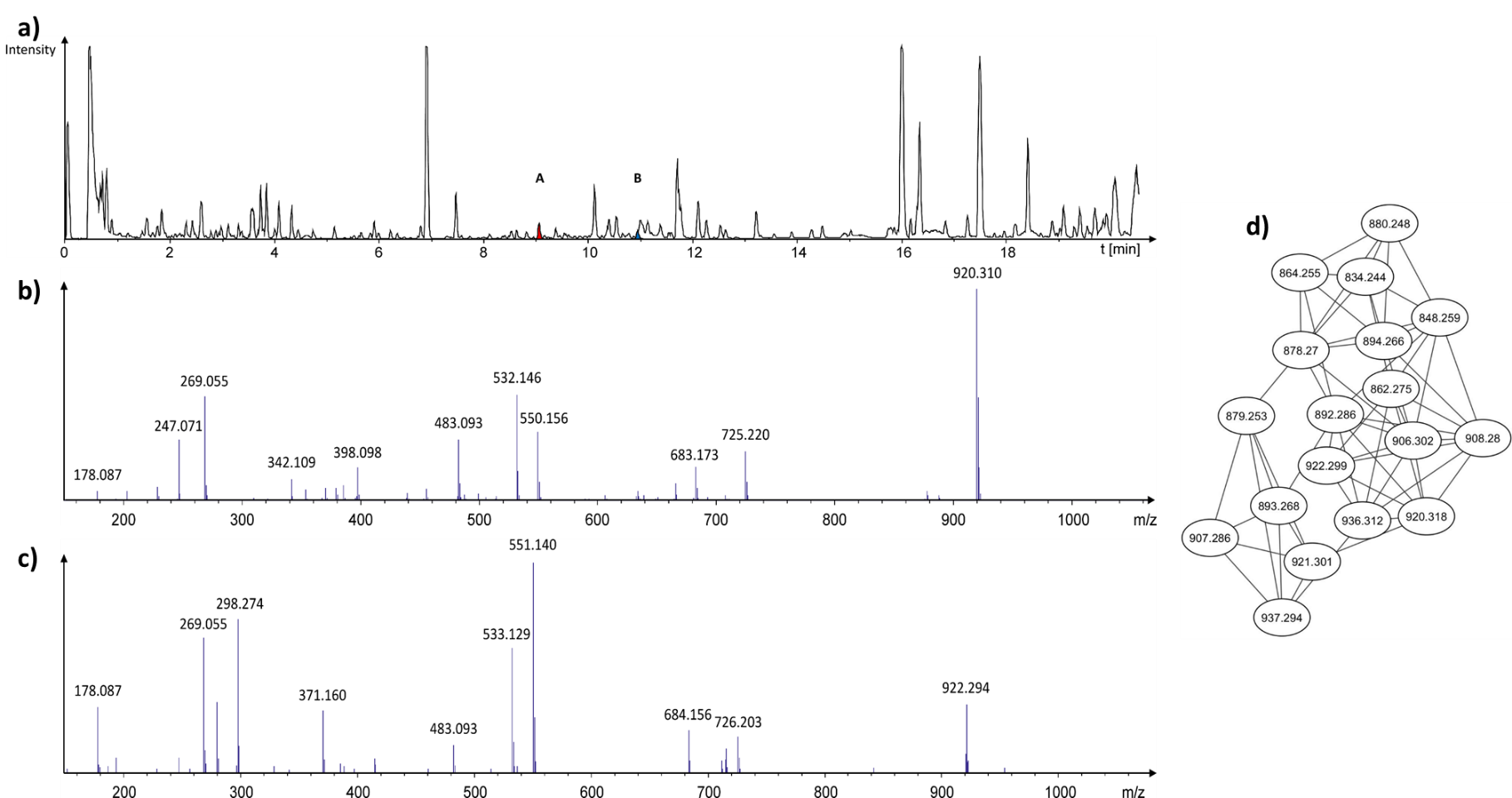


**Figure S6:** a) LC-MS Base peak chromatogram of the OMV extract (black) highlighting the two major cystobactamids: cystobactamid 919-1 (A, red, m/z 920.31 ± 0.05 Da) and cystobactamid 920-1 (B, blue, m/z 921.30 ± 0.05 Da) as extracted ion chromatograms. b) ESI-CID-MS² spectrum of cystobactamid 919-1 from the OMV samples measured on a maXis 4G qTOF spectrometer c) ESI-CID-MS² spectrum of cystobactamid 920-1 from the OMV samples measured on a maXis 4G qTOF spectrometer d) Spectral network created by GNPS using the MS² data from the OMV extract samples depicting the precursor masses of all cystobactamid derivatives contained in the samples.


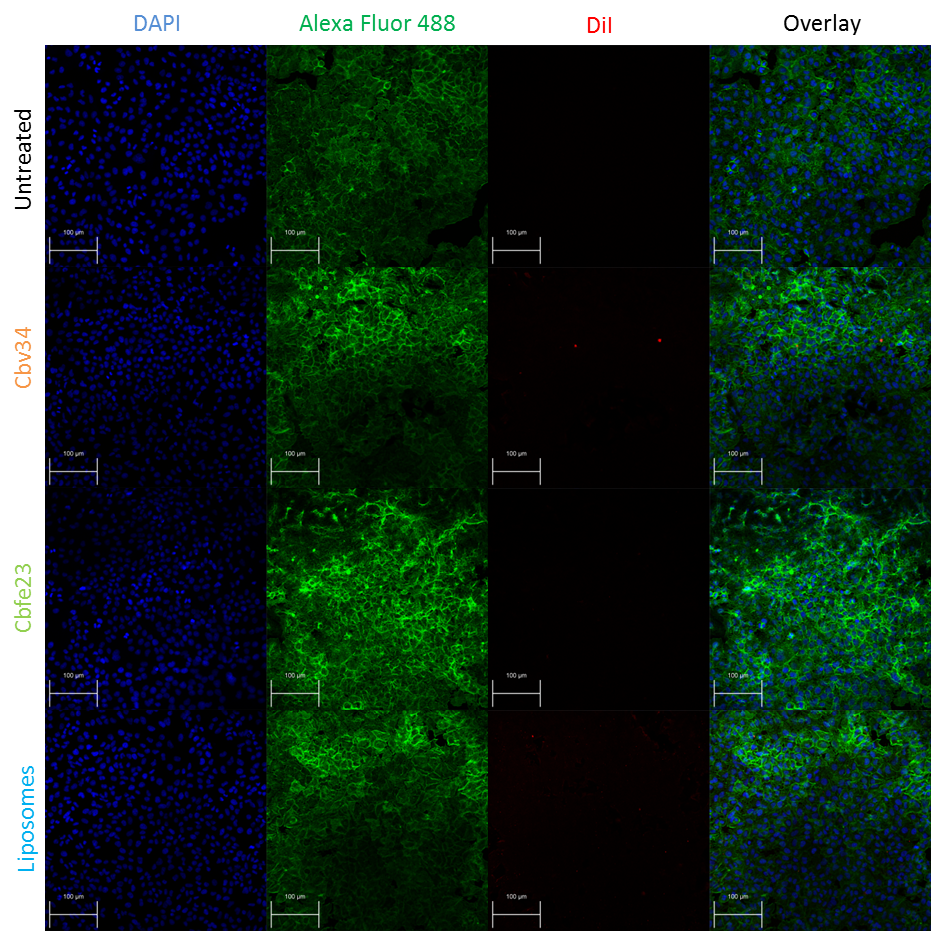


**Figure S7:** Uptake/interaction of OMVs and liposomes with A549 cells after 4 h incubation measured by confocal laser scanning microscopy. The cells nuclei are stained by DAPI (blue), while the F-actin is labelled by Alexa Fluor® 488 Phalloidin (green). Scale bars = 100 µm.


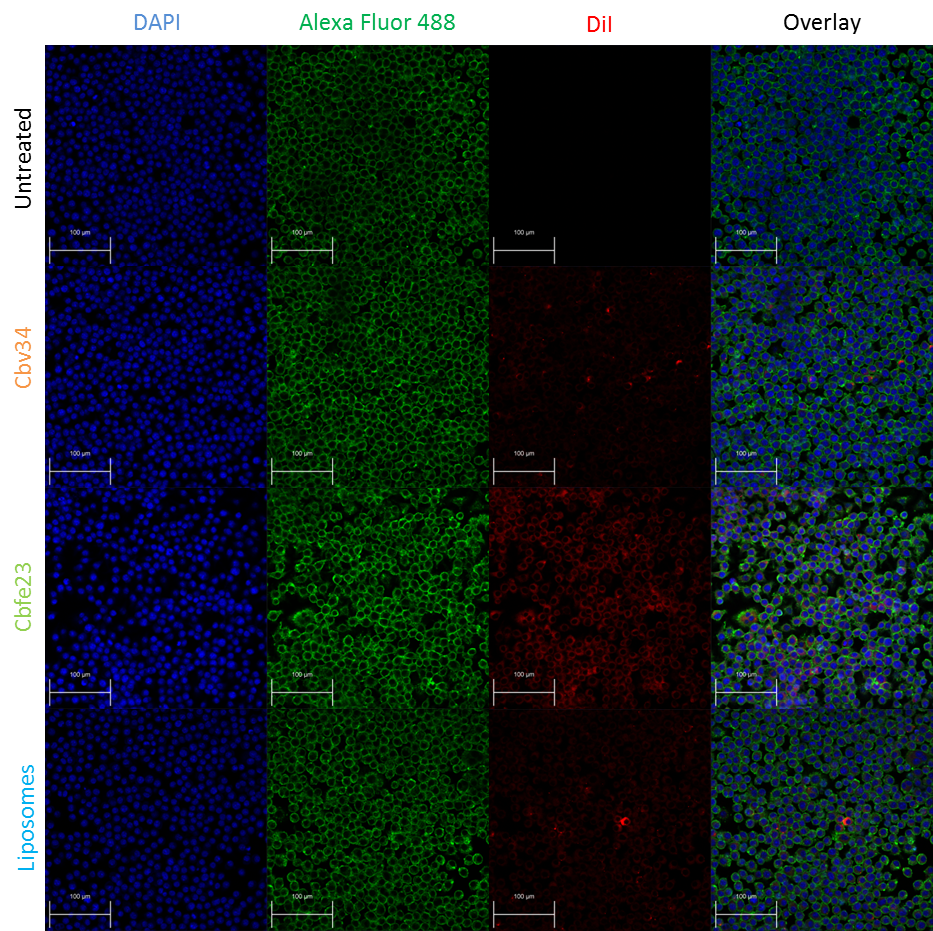


**Figure S8:** Uptake/interaction of OMVs and liposomes with RAW 264.7 cells after 4 h of incubation measured by confocal laser scanning microscopy. The cells nuclei are stained by DAPI (blue), while the F-actin is labelled by Alexa Fluor® 488 Phalloidin (green). Scale bars = 100 µm.


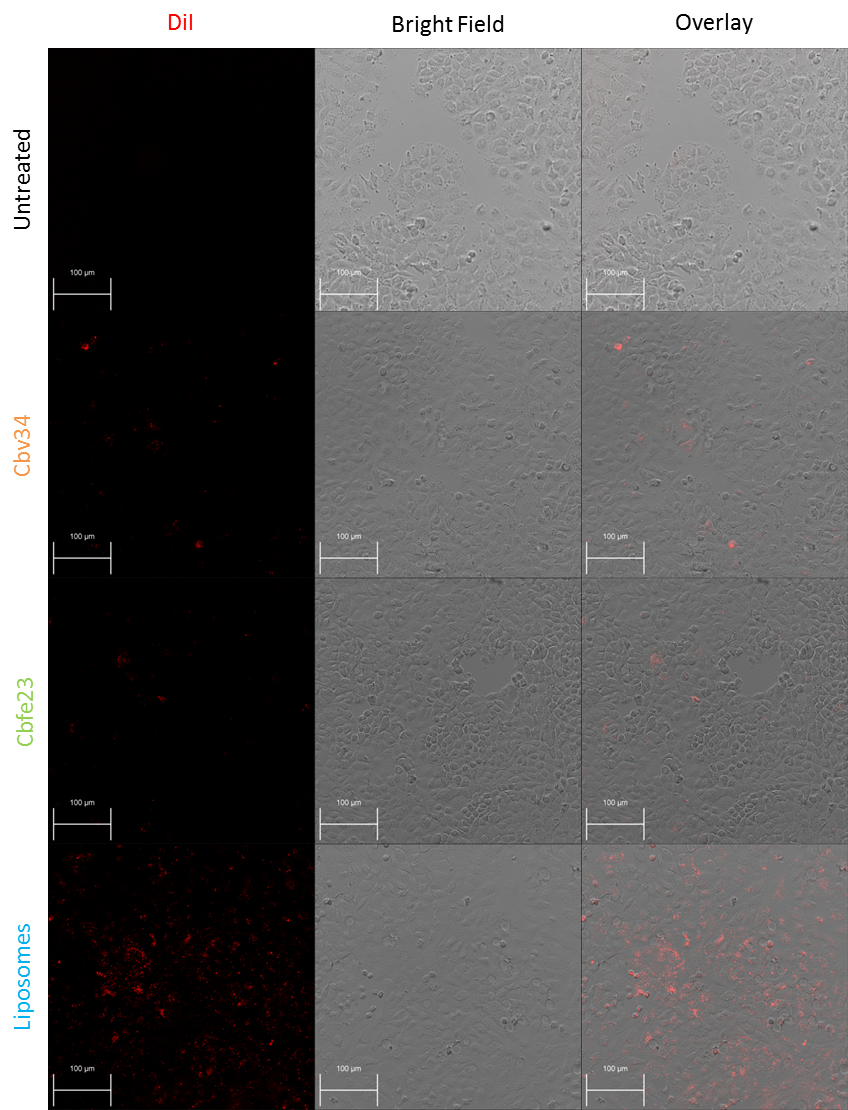


**Figure S9:** Uptake/interaction imaging of OMVs and liposomes after 4 h incubation with A549, following one single wash with PBS and fixation by confocal laser scanning microscopy. Cells were imaged with bright field and OMVs and liposomes were stained with DiI (red). Scale bars = 100 µm.


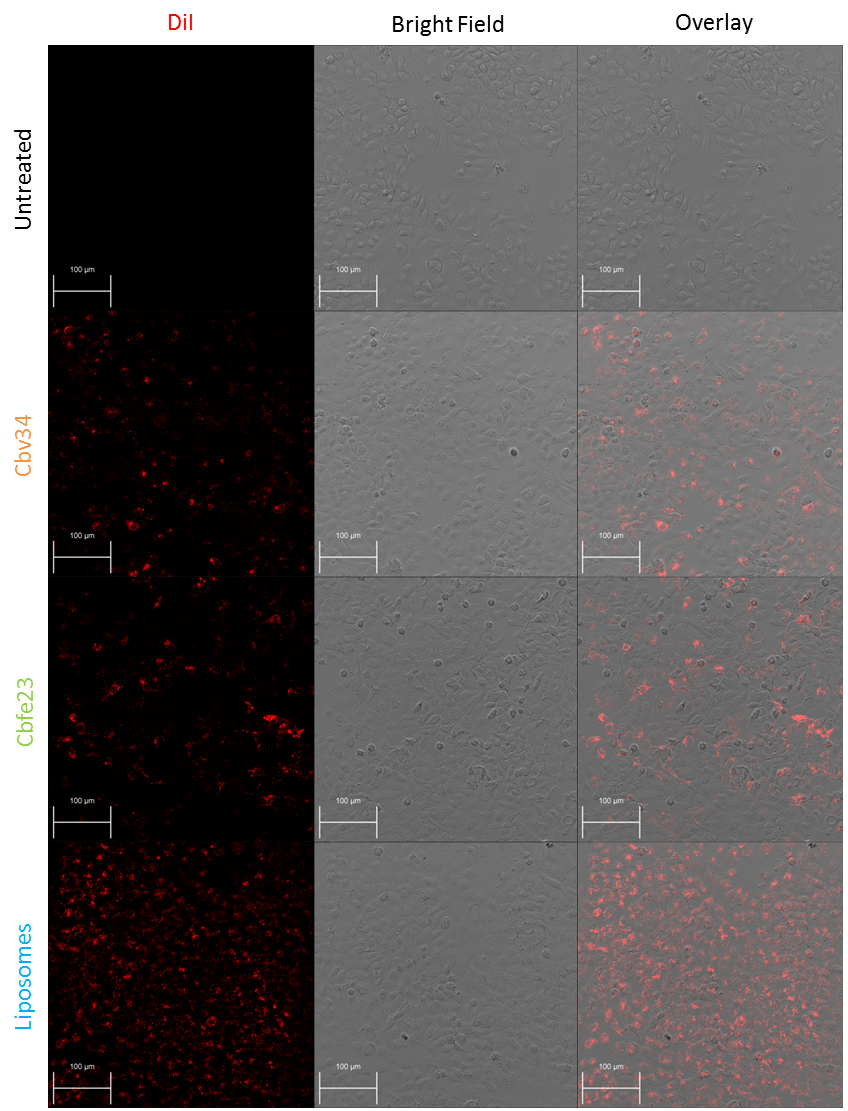


**Figure S10:** Uptake/interaction imaging of OMVs and liposomes after 24 h incubation with A549, following one single wash with PBS and fixation by confocal laser scanning microscopy. Cells were imaged with bright field and OMVs and liposomes were stained with DiI (red). Scale bars = 100 µm.
